# 1549. Risk Factors, Risk Perception, and Long-acting PrEP Awareness and Interest among US Women: A National Survey

**DOI:** 10.1093/ofid/ofad500.1384

**Published:** 2023-11-27

**Authors:** Tonia Poteat, Supriya Sarkar, Leigh Ragone, Danielle Bogan, Kyli Gallington, Karin S Coyne, Patrick Daniele, Keith Rawlings, Alex R Rinehart, Vani Vannappagari

**Affiliations:** Duke University School of Nursing, North Carolina; ViiV Healthcare, Durham, North Carolina; ViiV Healthcare, Durham, North Carolina; Viiv Healthcare, Durham, North Carolina; Evidera, Sacramento, California; Evidera, Sacramento, California; Evidera, Sacramento, California; ViiV Healthcare, Durham, North Carolina; ViiV Healthcare, Durham, North Carolina; ViiV Healthcare, Durham, North Carolina

## Abstract

**Background:**

Cisgender women account for approximately 20% of incident HIV diagnoses in the US. However, only 10% of women who could benefit from HIV pre-exposure prophylaxis (PrEP) have been prescribed PrEP. US women face several challenges that may affect their seeking HIV prevention services, such as lack of PrEP awareness. Understanding these challenges may improve PrEP uptake, especially as innovative PrEP options, such as long-acting (LA) injectable PrEP, become available. This study explored interest in using LA-PrEP by factors that may increase risk of HIV acquisition.

**Figure:**
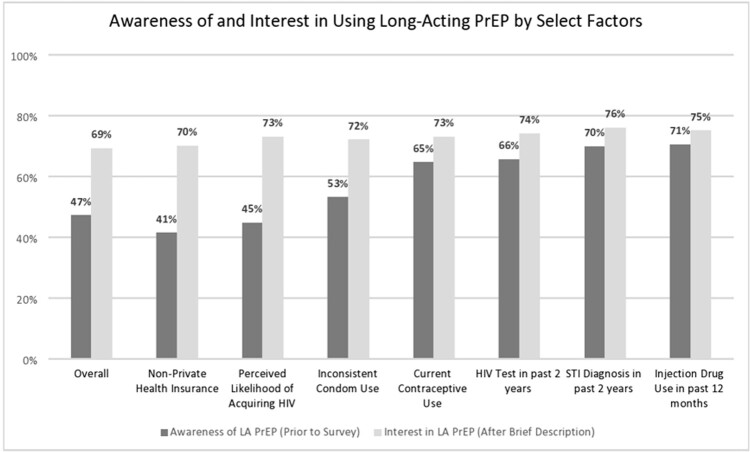

**Methods:**

Cisgender women were recruited between November 2021 – March 2022 though targeted social media ads on Facebook, Instagram, and Tinder to complete an online, self-administered survey. Eligible participants were cisgender women who were: 18+ years, current US residents, reported penetrative sex in the past six months, and reported an unknown or negative HIV status. The survey included questions on demographics, sexual health and behavior, LA-PrEP awareness, and LA-PrEP interest. Descriptive statistics were calculated using SAS v9.4.

**Results:**

1,834 eligible women completed the survey (median age: 28 years (IQR: 25, 33); Geography: South – 65%, West – 21%, Northeast – 7.3%, Midwest – 6.8%; Race: Black, non-Hispanic – 34%, White, non-Hispanic – 25%, Hispanic – 27%, Another race – 14%). Women were more likely to have heard of LA-PrEP if they had reported injection drug use in the past year (71%), had an STI diagnosis (70%) or HIV test (66%) in the past two years, and were currently using contraception (65%). Interest in using LA-PrEP was consistently high; the difference between LA-PrEP awareness and interest was greatest among women with non-private health insurance and those with perceived likelihood of future HIV acquisition.

**Conclusion:**

Although interest in using LA-PrEP is high among US women, these results demonstrate the large gap between LA-PrEP awareness and interest in using it.

**Disclosures:**

**Tonia Poteat, PhD, MPH, PAC**, Viiv Healthcare: Advisor/Consultant **Supriya Sarkar, PhD, MPH**, GlaxoSmithKline: Stocks/Bonds|Viiv Healthcare: Employee **Leigh Ragone, MS**, GlaxoSmithKline: Stocks/Bonds|ViiV Healthcare: Employee **Danielle Bogan, BSN, MPH,DrPH**, ViiV Healthcare: Contract worker **Keith Rawlings, MD**, GlaxoSmithKline: Stocks/Bonds|ViiV Healthcare: Employee **Alex R. Rinehart, PhD**, ViiV Healthcare: Employee **Vani Vannappagari, MBBS, MPH, PhD**, GlaxoSmithKline: Stocks/Bonds|ViiV Healthcare: Employee

